# Feasibility of Conducting Long-term Health and Behaviors Follow-up in Adolescents: Longitudinal Observational Study

**DOI:** 10.2196/37054

**Published:** 2022-08-15

**Authors:** Giovanni Cucchiaro, Luis Ahumada, Geoffrey Gray, Jamie Fierstein, Hannah Yates, Kym Householder, William Frye, Mohamed Rehman

**Affiliations:** 1 Johns Hopkins All Children's Hospital St. Petersburg, FL United States

**Keywords:** Fitbit, wearables, health tracker, survey, adolescents, psychosocial, long term, follow-up, feasibility, artificial intelligence, machine learning, posterior spine fusion, operation, surgery

## Abstract

**Background:**

Machine learning uses algorithms that improve automatically through experience. This statistical learning approach is a natural extension of traditional statistical methods and can offer potential advantages for certain problems. The feasibility of using machine learning techniques in health care is predicated on access to a sufficient volume of data in a problem space.

**Objective:**

This study aimed to assess the feasibility of data collection from an adolescent population before and after a posterior spine fusion operation.

**Methods:**

Both physical and psychosocial data were collected. Adolescents scheduled for a posterior spine fusion operation were approached when they were scheduled for the surgery. The study collected repeated measures of patient data, including at least 2 weeks prior to the operation and 6 months after the patients were discharged from the hospital. Patients were provided with a Fitbit Charge 4 (consumer-grade health tracker) and instructed to wear it as often as possible. A third-party web-based portal was used to collect and store the Fitbit data, and patients were trained on how to download and sync their personal device data on step counts, sleep time, and heart rate onto the web-based portal. Demographic and physiologic data recorded in the electronic medical record were retrieved from the hospital data warehouse. We evaluated changes in the patients’ psychological profile over time using several validated questionnaires (ie, Pain Catastrophizing Scale, Patient Health Questionnaire, Generalized Anxiety Disorder Scale, and Pediatric Quality of Life Inventory). Questionnaires were administered to patients using Qualtrics software. Patients received the questionnaire prior to and during the hospitalization and again at 3 and 6 months postsurgery. We administered paper-based questionnaires for the self-report of daily pain scores and the use of analgesic medications.

**Results:**

There were several challenges to data collection from the study population. Only 38% (32/84) of the patients we approached met eligibility criteria, and 50% (16/32) of the enrolled patients dropped out during the follow-up period—on average 17.6 weeks into the study. Of those who completed the study, 69% (9/13) reliably wore the Fitbit and downloaded data into the web-based portal. These patients also had a high response rate to the psychosocial surveys. However, none of the patients who finished the study completed the paper-based pain diary. There were no difficulties accessing the demographic and clinical data stored in the hospital data warehouse.

**Conclusions:**

This study identifies several challenges to long-term medical follow-up in adolescents, including willingness to participate in these types of studies and compliance with the various data collection approaches. Several of these challenges—insufficient incentives and personal contact between researchers and patients—should be addressed in future studies.

## Introduction

Physicians and public health workers are often asked to make predictions on patient health outcomes after specific medical and surgical treatments. Over the years, many medical specialties have created prediction models to identify treatments that optimize patient health outcomes [1,2]. In general, these systems are based on traditional logistic regression models that use a limited set of clinical and physiological variables.

Machine learning (ML) uses statistical learning to uncover complex nonlinear relationships among data. ML identifies patterns from big data sets and, subsequently, enables researchers to predict outcomes [3]. Discovering nonlinearities in the data is how ML techniques such as neural networks can complement traditional statistical methods for analyzing complex medical problems, such as presurgical, intrasurgical, and postsurgical outcomes [4].

Recent advances in wearable technology, as well as improvements in data mining capability, have allowed physicians to gain access to a wide variety of patients’ physiologic, behavioral, and psychological data that can be used to build ML models [5,6]. Wearable technology enables the repeated or continuous digital measurement of different types of parameters. Typically, the real-time measurement of the 4 basic vital signs (ie, temperature, heart rate, respiration rate, and blood pressure) is limited to a single event over several months, most often during a visit to a primary care physician. Wearables enable the longitudinal measurement of these and other parameters with high precision [7]. Similarly, wearables can provide approximate information about sleep patterns, differentiating wake from sleep [8], as well as accurately measuring step count and activity duration [9].

There are few studies evaluating the feasibility of the various data collection methods in this context. There are still substantial barriers to the widespread use of wearable technology and web-based surveys in clinical research among adolescents. Although most of the technological barriers have been addressed in the last decade, patients’ compliance with wearing the tracking devices as well as patients’ fatigue from repeated communications with the care team are still major challenges. Reports from commercial studies indicate that 50% of new users of wearables and 74% of new users of health apps stop using them within 2 weeks [10]. Patient participation is often related to the extent of how they feel the clinical study they initially agree to participate in can actually meet their needs, fulfill their expectations, and align with their goals [11,12]. In general, patient attitude is often tied to perceived usefulness in long-term studies [13].

Given these considerations, this study evaluated the feasibility of monitoring health parameters in adolescents prior, during, and 6 months after a posterior spine fusion (PSF) operation. We assessed patients’ adherence with wearing a health tracking device, answering web-based surveys, and filling out paper surveys. We also evaluated the feasibility of data extraction from the hospital data warehouse and the aggregating of the extracted data with data from the original electronic medical record (EMR).

## Methods

### Recruitment and Study Population

Patients were approached at the time of their evaluation in the orthopedic surgeons’ office once they had been scheduled for a PSF operation and evaluated to determine whether they met the inclusion criteria, which included the following.

- Patients with idiopathic scoliosis scheduled for PSF surgery at Johns Hopkins All Children’s Hospital
- Patients of both sexes, aged 12 to 19 years
- Participants, parents, or legal guardians with reliable Wi-Fi or a cellular internet access data plan
- Participants, parents, or legal guardians with a compatible Bluetooth smartphone or home computer
- Completion of written informed consent by participants, their parents, or legal guardians
- Participants and parents were able to understand the instructions to upload data

Once the consent to participate in the study was obtained, we distributed a Fitbit device with the corresponding instructions on its use and downloaded the information onto a secure website for subsequent data analysis.

### Data Sources and Study Variables

Data were collected from the following sources: (1) wearable devices (Fitbit Charge 4), (2) hospital data warehouse, (3) electronic surveys, and (4) paper pain diaries.

#### Adherence

We defined adherence to the protocol as patients who answered at least 75% of the Qualtrics surveys and wore the tracking device at least 75% of the time.

#### Fitbit Data

Patients received a Fitbit Charge 4 (Fitbit) at the time of enrollment. The Fitbit Charge 4 is a lightweight, noninvasive wearable tracking device that reports daily step count, vigorous activity minutes, heart rate, and sleep patterns to the user’s smartphone or computer. The study team asked the patients to sync the device on a daily basis. We created personal Fitbit accounts linked to Fitabase (Small Steps Labs), a Health Insurance Portability and Accountability Act–compliant, encrypted, and password-protected portal that aggregates data from the Fitbit server for easier extraction.  We collected the following data from Fitabase: daily steps, time spent asleep (minutes/day), and heart rate (beats/minute).  Data captured by the Fitbit were synced to the patient’s personal device using the Fitbit app and subsequently stored on the central cloud-based Fitbit cloud services. The Fitabase portal extracted the patient-level data from the Fitbit cloud services.

GPS function was disabled by default by the study coordinators, and study participants were advised not to alter this functionality on their device or smartphone app.   

#### EMR Data

A variety of demographic and physiologic data are stored in the EMR. Johns Hopkins All Children’s Hospital has built a physiologic data warehouse that stores inpatient physiologic data in a long-term storage solution. The system comprises data captured from operating rooms and specific patient care areas throughout the hospital. For the purpose of this study, we retrieved age, sex, medications, and pain scores. Once data were extracted from the hospital data warehouse, they were stored in a REDCap research database (REDCap Consortium). Data were collected before, during, and for 6 months after the PSF operation.

#### Psychosocial Data

To identify psychosocial difficulties or barriers to recovery from surgery, psychosocial surveys were administered to patients via the QualtricsXM web-based software platform (SAP America Inc) before, during, and 3 and 6 months post-PSF surgery. We used the following validated surveys: Pain Catastrophizing Scale [12], Patient Health Questionnaire [13], Generalized Anxiety Disorder Scale [14], and Pediatric Quality of Life Inventory [15]. The survey data were collected and stored using REDCap.

#### Pain Diary Data 

Patients completed a daily paper diary documenting pain levels and analgesic medications. These data were manually inputted and stored in REDCap. 

### Statistical Analysis

Continuous variables were summarized with means, SDs, and ranges (minimum to maximum), and categorical variables were summarized with counts and percentages. Statistical comparisons between compliant and noncompliant groups were evaluated with 2-tailed *t* tests for independent samples, and 2-sided *P* values <.05 were considered statistically significant. Statistical analyses were performed using the *Pandas* and *NumPy* Python packages (Python Software Foundation).

### Ethics Approval

This study was reviewed and approved by the Johns Hopkins All Children’s Hospital Institutional Review Board (IRB00211758). Written consent was obtained from the parents and study participants.

## Results

In total, 2 orthopedic surgeons evaluated 108 patients with a diagnosis of idiopathic scoliosis between October 2020 and September 2021. Of the 108 patients scheduled for a PSF surgery, 84 (77.7%) were approached. Of the 84 patients approached, 32 (38%) were enrolled in the study. The reasons why we were not able to enroll the other patients approached are listed in Table 1.

Table 1 lists the reasons why 62% (52/84) of the patients who were approached were not enrolled in the study. Of the 52 patients not enrolled, 25% (n=13) did not meet the inclusion criteria. In 46% (n=24) of the patients, we were not able to reach out on time to enroll them or there was not sufficient time to establish at least 2 weeks of baseline values prior to the scheduled surgery because of staffing limitations.

At the time of writing, of the 32 enrolled patients, 13 (41%) have completed the 6-month follow-up period, and 3 (9%) are still in the process of completing the follow-up. The remaining 16 (50%) patients dropped out during the follow-up period and did not complete the study. Table 2 lists the reasons why patients did not complete the 6-month study period. Patients dropped out of the study on average 17.6 (SD 7.2; range 6-31) weeks from the day of surgery (Table 3). Of the 16 patients who dropped out of the study, the average age was 14 (SD 1.3; range 12-17) years, and there were 10 (62%) girls and 6 (38%) boys. Of the 16 patients who completed the study, there were 11 (69%) girls and 5 (31%) boys, and the average age was 14.8 (SD 1.9; range 13-18) years. There was no statistical difference between the 2 groups (*P*=.80) with respect to age and sex distribution. We defined noncompliant patients as those who did not complete the Qualtrics survey at least 75% of the time.

Of the 32 enrolled patients, 12 (38%) were considered noncompliant because they failed to complete the Qualtrics survey at least 75% of the time. However, 1 of them completed the Fitbit portion of the study. The remaining 11 patients stopped synchronizing the device within 1 to 5 months after the surgery.

Of the 13 patients who completed the follow-up, 12 (92%) patients were consistent in wearing the Fitbit at least 75% of the time during the day (Table 4). Their adherence was constant over the 6-month study. Additionally, 11 (85%) patients were also compliant in wearing the Fitbit at least 75% of the time at night for the duration of the study (Table 4).

Of the 13 patients who completed the follow-up, 9 (69%) completed the Qualtrics questionnaires throughout the entire study, and only 4 (31%) completed the Qualtrics questionnaires at least 75% of the time over the study period (Table 5).

The follow-up involved paper-based surveys to assess pain scores and the use of analgesic medications. None of the 13 patients who have completed the study were compliant with completing the pain paper diary during the study period.

We were able to retrieve the pain scores from the hospital data warehouse. These data were manually matched with those recorded in the patients’ EMR, and no discrepancies were found between the 2 databases. We collected additional demographic and clinical data, including the daily morphine equivalent doses of opioids administered and amount of blood transfusions (data not shown).

**Table 1:** Reasons why patients were not enrolled in the study.

Reason	Patient (N=84), n (%)	Detail
Not meeting inclusion criteria	13 (15)	Either above or below study age limits
Unable to operate Fitbit	5 (6)	Mental delay or Spanish-speaking only
Eligible for the study but denied participation	10 (12)	N/A^a^
Researchers missed enrollment encounter	19 (23)	Limited staffing
Not sufficient time to obtain baseline values	5 (6)	Changes in surgery scheduling

^a^N/A: not applicable.

**Table 2:** Reasons why patients dropped out of the study once they were enrolled.

Reason	Patient (N=32), n (%)
Completed study	13 (41)
Still enrolled	3 (9)
Parents asked to stop	2 (6)
Lost Fitbit	2 (6)
Noncompliant	12 (38)

**Table 3:** Duration of patients’ participation in the study before dropping out prior to the 6-month (26 weeks) follow-up. There was 1 patient who never started the study even though she signed the written consent form.

Patient number	Study participation (week), n
1	9
2	21
3	26
4	31
5	11
6	10
7	14
8	6
9	24
10	23
11	26
12	19
13	16
14	15
15	12
16	18

**Table 4:** Patients’ adherence with wearing Fitbit devices during the day and night. Data reflect the percent of time patients wore the Fitbit during the first and second study periods (1-3 and 4-6 months, respectively).

Study period, percentage of time wearing the Fitbit		During the day (N=13), n (%)		During the night (N=13), n (%)
**1-3 months**				
	100%	11 (85)	5 (38)	
	75%	1 (8)	6 (46)	
	50%	1 (8)	2 (15)	
	25%	0 (0)	0 (0)	
**4-6 months**				
	100%	7 (54)	6 (46)	
	75%	5 (38)	5 (38)	
	50%	1 (8)	1 (8)	
	25%	0 (0)	1 (8)	

**Table 5:** Number of patients who responded to the Qualtrics surveys over the study period.

Percentage response to Qualtrics surveys	Patient (N=13), n (%)
100%	9 (69)
75%	4 (31)
50%	0 (0)
25%	0 (0)

## Discussion

### Principal Findings

The goal of this study was to assess whether it is possible to collect meaningful and reliable clinical data over extended periods of time from adolescents who needed a surgical procedure using different techniques. This was the preliminary phase of a project that will use ML to predict outcomes after complex surgical procedures in children and adolescents.

This study showed that it is possible to conduct long-term health outcome assessment in adolescents using tracking devices and web-based apps that allow repeated surveys of populations. The study confirmed that there are multiple obstacles to conducting and completing this type of study. The willingness of adolescents and parents to participate and complete the surveys are major limiting factors. It was not surprising to find that adolescents were more compliant with completing electronic surveys than paper surveys.

### Comparisons With Prior Work

There are few data on children and adolescents’ willingness to participate in clinical trials as well as their use of tracking devices and electronic surveys.

We faced several challenges when conducting this study. We encountered problems enrolling patients in the study. Once patients were enrolled, there was a relative high number of dropouts. Only 38% of the patients we contacted agreed to be enrolled in the study, and once enrolled, only 50% of them completed the study.

Adult studies have shown that provider and patient factors are common barriers to patients’ enrollment in clinical studies. A lack of time and resources often prevent physicians from being involved in patients’ enrollment [14,15]. The presence of a strong, trusting relationship between physicians and patients is often a determining factor in patients and parents’ willingness to participate in a study [16]. The fear of experimentation and medical mistrust are common barriers to participation in clinical studies [17,18]. Privacy concerns may have also played a role in parents’ decision not to participate in the study. Our low number of enrolled patients might be attributed to the lack of direct contact between the principal investigator and patients, as patients were contacted by research personnel who were not directly involved in the patients’ care. Similarly, the lack of personal contact between the researchers and patients may have influenced the large number of dropouts.

The feasibility of using ML techniques is predicated on the access to patients’ data and building reliable data sets. We collected data prior, during, and after the hospital stay of adolescents who underwent a PSF operation. We used paper-based surveys, electronic surveys, wireless-enabled wearable technology, and hospital-based data warehouse to collect patients’ data. The goal was to acquire a broad set of data, including numerical, categorical, time-series, and text data.

Peer-reviewed research on the use of Fitbit devices found that the device is reliable for tracking daily activity in healthy young adults [19]. Barriers to wearing tracking devices have been reported in the literature. Many of the issues reported in early studies have been addressed by the manufacturer as new devices are now waterproof and new batteries provide long intervals between recharges. With respect to monitoring sleep, devices such as Fitbits are not a substitute of polysomnography. However, there is consensus among researchers that devices such as Fitbit can provide gross estimates of time spent sleeping [8]. It is still controversial whether they can offer an accurate reading of the sleep stages [20]. The new generation Fitbits can provide a much wider set of information.

We were able to retrieve the number of steps and the number of minutes the patients spent sleeping from the Fitabase.

It was interesting to notice that the patients who remained in the study for 6 months were also very compliant with wearing the Fitbit. The patients who were not compliant with answering the Qualtrics surveys were also not compliant with wearing the Fitbit, with the exception of 1 patient. This seems to indicate that they dropped out because of a general lack of interest in continuing with the study and not because of the specific burden of complying with either the Qualtrics survey or wearing the Fitbit.

A potential draw back on the long-term use of these tracking devises is the novelty factor, with waning interest in wearing these devices after a couple of weeks [21,22]. Our overall patients’ adherence with constantly wearing the Fitbit was similar to their adherence with answering the Qualtrics surveys. Further analysis and possibly creating focus groups will help the understanding of these differences in adolescents’ behaviors.

The widespread introduction of EMRs has allowed clinician, administrators, and researchers to have rapid access to a wealth of patients’ demographic and clinical data. EMR data can be transferred into clinical and research registers independently from registry purposes. We were able to collect data we consider to be relevant in this pilot feasibility study. There are challenges to this methodology, with the main concern being the accuracy of the data inputted into the EMR and registries. Authors have suggested creating some form of data curation to review and assess data quality. For the purpose of this study, we collected demographic and numerical data while the patients were hospitalized, with the majority of the data automatically recorded by hospital monitor devices. The only self-reported data were the pain scores, where nurses used a numeric scale to document the patients’ pain levels during the hospital stay [23]. We confirmed the accuracy of the process by manually verifying the EMR with the data extrapolated from the hospital warehouse.

It was not surprising to observe a low adherence to filling out the follow-up paper diary documenting the patients’ pain level and use of analgesic medications. Recent surveys have shown that approximately 75% of adolescents own a smartphone [24]. The same survey highlighted how teenagers prefer texting to talking to their peers. In addition, there is also some evidence that adolescents prefer electronic books to paper books [25]. These findings may explain why our patients were compliant with answering electronic surveys but did not complete the paper surveys. Additionally, a very demanding schedule where patients were asked to report daily pain scores and medications used for 6 months may have caused survey fatigue.

### Limitations

There are a few limitations to this study. The relatively low percentage of patients willing to participate and then comply with the study requirements may have introduced a selection bias. The lack of data on pain levels throughout the study has resulted in the inability to make correlations between pain levels and behaviors.

### Conclusions

This preliminary study showed that it is possible to conduct long-term studies and evaluate the different aspects of adolescents’ behaviors and outcomes of health interventions using wearable devices and web-based surveys. These technologies may substantially lower the cost of conducting this type of research.

This study confirmed that there are barriers to engaging relatively healthy adolescents and keeping them interested in participating in this type of long-term study.

Further studies are needed to identify better ways of informing patients and their families of the relevance of these long-term studies. Researchers should also design strict reminder schedules including periodic texts, phone calls, and emails and consider rewards to keep patients engaged in these long-term surveys.

## Abbreviations

EMR: electronic medical record
ML: machine learning
PSF: posterior spine fusion
